# Prevalence and correlates of adherence to movement guidelines among urban and rural children in Mozambique: a cross-sectional study

**DOI:** 10.1186/s12966-019-0861-y

**Published:** 2019-10-28

**Authors:** Taru Manyanga, Joel D. Barnes, Jean-Philippe Chaput, Peter T. Katzmarzyk, Antonio Prista, Mark S. Tremblay

**Affiliations:** 1 Healthy Active Living and Obesity Research Group, CHEO Research Institute, 401 Smyth Road, Ottawa, ON K1H 8L1 Canada; 2 Faculty of Medicine, School of Epidemiology and Public Health, University of Ottawa, Ottawa, Canada; 3 Pennington Biomedical Research Center, Baton Rouge, Louisiana, USA; 4 Research Group for Physical Activity and Health (CIDAF-FEFD) Universidade Pedagógica, Maputo, Mozambique

**Keywords:** 24-h movement guidelines, Correlates, Urban/rural comparison, Moderate- to vigorous-intensity physical activity, Sleep, Recreational screen time

## Abstract

**Background:**

Insufficient physical activity, short sleep duration, and excessive recreational screen time are increasing globally. Currently, there are little to no data describing prevalences and correlates of movement behaviours among children in low-middle-income countries. The few available reports do not include both urban and rural respondents, despite the large proportion of rural populations in low-middle-income countries. We compared the prevalence of meeting 24-h movement guidelines and examined correlates of meeting the guidelines in a sample of urban and rural Mozambican schoolchildren.

**Methods:**

This is cross-sectional study of 9–11 year-old children (*n* = 683) recruited from 10 urban and 7 rural schools in Mozambique. Moderate- to vigorous-intensity physical activity (MVPA) and sleep duration were measured by waist-worn Actigraph GT3X+ accelerometers. Accelerometers were worn 24 h/day for up to 8 days. Recreational screen time was self-reported. Potential correlates of meeting 24-h movement guidelines were directly measured or obtained from validated items of context-adapted questionnaires. Multilevel multivariable logit models were used to determine the correlates of movement behaviours. Meeting 24-h movement guidelines was defined as ≥60 min/day of MVPA, ≤2 h/day of recreational screen time, and between 9 and 11 h/night of sleep.

**Results:**

More rural (17.7%) than urban (3.6%) children met all three 24-h movement guidelines. Mean MVPA was lower (82.9 ± 29.5 min/day) among urban than rural children (96.7 ± 31.8 min/day). Rural children had longer sleep duration (8.9 ± 0.7 h/night) and shorter recreational screen time (2.7 ± 1.9 h/day) than their urban counterparts (8.7 ± 0.9 h/night and 5.0 ± 2.3 h/day respectively). Parental education (OR: 0.37; CI: 0.16–0.87), school location (OR: 0.21; CI: 0.09–0.52), and outdoor time (OR: 0.67; CI: 0.53–0.85) were significant correlates of meeting all three 24-h movement guidelines.

**Conclusions:**

Prevalence and correlates of meeting movement guidelines differed between urban and rural schoolchildren in Mozambique. On average, both groups had higher daily MVPA minutes, shorter sleep duration, and higher recreational screen time than the 24-h movement guidelines recommend. These findings (e.g., higher than recommended mean daily MVPA minutes) differ from those from high-income countries and highlight the need to sample from both urban and rural areas.

## Introduction

Insufficient physical activity (PA) [1], inadequate sleep duration [2], and excessive recreational screen time (ST) [3] have been linked with a higher risk of childhood obesity, cardiovascular disease, metabolic syndrome, type 2 diabetes mellitus, and all-cause mortality in adulthood [4–7]. Surveillance mechanisms for the prevention and identification of those most at risk for the health consequences associated with suboptimal MVPA or sleep includes national [8, 9] and global [10, 11] 24-h movement guidelines for children and youth. Although there are currently no quantitative guidelines for total sedentary behaviours, some countries have developed and adopted guidelines for maximum daily ST as one dimension of sedentary behaviour [8, 9]. 24-Hour Movement Guidelines for Children and Youth [8–10] recommend that within a 24-h period, children and youth should accumulate at least 60 min of MVPA, engage in no more than 2 h of ST, and obtain uninterrupted sleep of 9 to 11 h per night. For the present study, participants were classified as meeting the overall guidelines if they met all three recommendations.

Research evidence show that insufficient PA [1], short sleep duration [2], and excessive ST [3] among children are reaching alarming levels globally. For example, in 2010, self-reported data from the Global Health Observatory showed that 81% of 11–17 year-old youth did not accumulate the recommended ≥60 min of daily moderate- to vigorous-intensity physical activity (MVPA) [12]. A recent review showed that overall, 5–18 year-old children and adolescents from 20 countries have been losing an average of 0.76 min of sleep/year over the past century [2], although the evidence for secular trends is mixed, conflicting and limited [13]. Evidence has also shown that 2–18 year-old children and adolescents spent 3–8 h in ST/day [3], while another review showed that 5–18 year-olds spent 51% of their off-school time in ST [14].

Currently, there are little to no data describing prevalences and correlates of movement behaviours among children in low-middle-income countries (LMICs) [15]. The few available reports do not include both urban and rural respondents, despite the large proportion of rural populations in LMICs [16]. For example, in Mozambique the little available objective evidence [17] documenting the prevalence of movement behaviours does not compare urban and rural children despite the well-known differences in their lifestyles. A study by Ojiambo et al. showed higher MVPA and lower sedentary behaviour among rural compared to urban Kenyan children [18]. Onywera et al. demonstrated that on average, rural Kenyan children accumulated significantly more step counts per day than those from urban areas [19]. In addition, systematic review evidence of studies from sub-Saharan Africa showed that children living in urban areas were more likely to engage in lower levels of PA, and higher sedentary behaviours than their rural counterparts [20]. The relative lack of current and robust evidence on movement behaviours was identified as an urgent priority in the most recent synthesis [21] of available data in Mozambique and highlighted in the recent World Health Organization Global Action Plan for Physical Activity [22]. Furthermore, research has shown that PA across the life-course can be affected by childhood PA behaviours and experiences [23, 24].

Results from previous studies [25–29] were used to identify potential correlates of 24-movement guidelines for the current study. The purpose of the present study was to 1) compare the prevalence of urban and rural school children meeting 24-h movement guidelines, and 2) examine correlates of meeting the 24-h movement guidelines in a sample of urban and rural Mozambican children. Given the available systematic review evidence [20], and the results from the studies by Ojiambo et al. [18] and Onywera et al. [19] in Kenya, where contexts are similar to those of Mozambique, we hypothesized that children attending rural schools would accumulate more minutes of MVPA, have longer sleep duration and less ST compared to those from urban schools and that correlates of movement behaviours would differ between urban and rural children.

## Methods

### Study design and participants

Participants (9–11 year-old primary schoolchildren) for this cross-sectional study (*n* = 683) were recruited from 10 urban (Maputo, stratified by socioeconomic status) and 7 rural (Macia district) schools in Mozambique. At least three urban schools were randomly recruited from each of three districts, using a list provided by the Ministry of Education to maximize variability in levels of neighborhood socioeconomic status (SES). Rural schools were conveniently recruited from a list provided by the district education office. One urban school declined to participate after being approached and was replaced by another school from the same district. Participants whose parents provided consent and were between ages 9 and 11 years old were included in the study. We followed the published protocol and methodology used in the International Study of Childhood Obesity, Lifestyle and the Environment (ISCOLE) [30]. Data were collected between August 2017 and May 2018. We adhered to the Strengthening the Reporting of Observational Studies in Epidemiology (STROBE) guidelines for cross-sectional studies (Additional file 1: Table S1).

#### Measurement of movement behaviours

### Sleep and MVPA

Actigraph GT3X+ accelerometers were used to objectively measure nocturnal sleep and PA. The accelerometers were attached to an elastic belt and worn over the right hip, for seven consecutive days plus an initial familiarization day. To improve compliance, a 24-h protocol was implemented, encouraging participants to wear the accelerometer at all times except during water-based activities [31]. Because of the 24-h accelerometer protocol, it was necessary to distinguish the nocturnal sleep episode from periods of awake non-wear time. This was done using a 60-s epoch and published algorithms [31, 32] that were developed for ISCOLE. After accounting for nocturnal sleep episode time, waking non-wear time was considered as any sequence of ≥20 consecutive minutes of zero activity counts [33]. Data were collected at a sampling rate of 80 Hz [30], downloaded in 1-s epochs [33] with the low-frequency extension filter using the ActiLife software version 6.5.4 (ActiGraph LLC, Pensacola, FL, USA). Consistent with the Evenson [34] and SADEH [35] criteria for summarizing PA and nocturnal sleep respectively, data were subsequently aggregated to 15- and 60-s epochs. After accounting for the total nocturnal sleep and awake non-wear time [32], all remaining minutes were classified as awake-wear time. Participants with ≥10 h of wear time/day on at least 4 days including 1 weekend day were considered to have sufficient PA data [30, 33]. Cut-points developed by Evenson et al. [34] were used to quantify MVPA (≥574 counts/15-s). Sleep duration and MVPA were dichotomized as those who did not meet the sleep duration guideline (< 9 h/night vs. meeting 9 to 11 h/night) and not meeting the MVPA guideline (< 60 min/day vs. meeting ≥60 min/day). Four participants (0.6%) had sleep durations between 11 and 11.5 h per night. These participants were considered to be meeting the sleep duration guideline, consistent with the recommended upper limit of up to 12 h per night for nightly sleep duration for school-aged children [10].

### Recreational screen time

As was done in ISCOLE, the *Demographic and Lifestyle Questionnaire* with questions derived from the U. S Youth Risk Behaviour Surveillance System was used to obtain data on ST [30]. Participants were asked the following questions: 1) on a school day, how many hours did you watch TV; 2) on a school day, how many hours did you play video or computer games or use a computer for something that was not school; 3) on a weekend day, how many hours did you watch TV; or 4) on a weekend day, how many hours did you play video or computer games or use a computer for something that was not school work? Response options were: did not watch TV, ≤ 1 h of TV, 2 h, 3 h, 4 h, and ≥ 5 h of TV. A weighted mean score of hours of daily ST was calculated as follows: [(hours of TV on weekdays × 5) + (hours of TV on weekend days × 2) + (hours of video games and computers on weekdays × 5) + (hours of video games and computers on weekend days × 2)]/7. Quantifying ST using self-reported methods has been shown to have acceptable reliability and validity in children [36]. ST was dichotomized as those who did not meet the ST guideline (> 2 h/day) versus those who met the guidelines (≤2 h/day).

### Correlates of meeting 24-h movement guidelines

Lifestyle and environmental factors associated with movement behaviours were obtained from validated items of previously published questionnaires [30] that were successfully used in ISCOLE. Questionnaires were forward and backward translated, and specific items on the questionnaires were adapted to reflect local contexts.

### Covariates

Participants’ sex and age, highest level of parental education (a proxy of socioeconomic status), and school location (urban/rural) were used as covariates in all multivariable models because of the plausibility of confounding.

### Statistical analyses

Statistical analyses were computed using SAS 9.4 (SAS Institute Inc., North Carolina, USA) and R (version 3.5.2; The R Foundation for Statistical Computing, Vienna, Austria). Descriptive characteristics of participants were summarized using means (SD) or frequencies (percentages) as appropriate. Unpaired t-tests (continuous variables) and chi-square tests (χ^2^) (categorical variables) were used to examine potential differences between participants attending urban versus rural schools. Multilevel multivariable logit models (PROC GLIMMIX) accounting for clustering at the school level were used to determine the correlates of meeting MVPA, sleep, ST, and MVPA + sleep + ST guidelines (0, no; 1, yes). Schools were treated as random effects in all models. For intercept estimates to be more meaningful, all continuous predictors were grand mean centered prior to estimating the models. Denominator degrees of freedom pertaining to fixed effects were calculated using the Kenward Roger approximation (DDFM = KR) [37]. Variance tolerance inflation factors, (VIF), condition index, and tolerance were applied to test for multicollinearity in multivariable models [38]. VIFs > 10, condition index > 30, and tolerance values less than 0.1 were considered to be indicative of potential multicollinearity threats [38]. The lowest tolerance value (0.785), highest VIF (1.274) and condition index (1.700) suggested no threats of multicollinearity.

Twenty-eight potential correlates of meeting movement guidelines were selected a priori, based on availability and plausibility of relationships with movement behaviors as per previous literature [25–29]. Correlates included directly measured and reported variables obtained from questionnaire data. Table 1 presents the list of the potential correlates and how they were used in the analyses. First, each potential correlate was included in univariable models and those that were at least marginally (*p* < 0.10) statistically significant were retained for use in the multivariable models. This less-strict criterion was applied for univariable analyses to prevent the potential exclusion of important variables. Potential correlates that remained marginally statistically significant (*p* < 0.10) from the univariable analyses were entered in final models including all variables and covariates. Variables that were statistically significant (*p* < 0.05) in the final models were considered to be correlates of meeting movement guidelines.

**Table 1:** Potential correlates of meeting movement guidelines

Variable	Method of measurement	Use in analysis
Potential correlates common to MVPA, Sleep duration, and ST.		
Sex	Parent-reported	Binary variable: male or female (covariate)
Age	Parent-reported	Continuous (covariate)
BMI z-score	Objectively measured	Continuous
School commute (mode of transport to and from school for main part of the journey)	Participant-reported	Re-coded as dichotomous: active (walking, bicycle / rollerblade / skateboard / scooter), or passive (bus / train / boat / car / motorcycle / moped)
Outdoor time (before school, after school, weekend)	Participant-reported	Continuous
Participation in sports	Participant-reported	Re-coded as dichotomous: (did not participate in sporting activities) or (participated in sporting activities) in the past year
Self-perceived health	Participant-reported	Re-coded as dichotomous: (poor, fair) or (good, very good, excellent)
Parental level of education	Parent-reported	Re-coded as highest level of parental education (covariate): <high school, high school/some college, or bachelor’s/graduate degree
Mother’s work status	Parent-reported	Continuous: re-coded as dichotomous (≤15 h/week) or (> 15 h/week)
Father’s work status	Parent-reported	Continuous: recoded as dichotomous (≤15 h/week) or (> 15 h/week)
School location	School-Administrator-reported	Binary: urban or rural (covariate)
Number of televisions in the house	Parent-reported	Continuous: re-coded as categorical: 0 or 1 or ≥ 2
Number of functional cars at home	Parent-reported	Continuous: re-coded as categorical: 0 or 1 or ≥ 2
Number of siblings for participant	Parent-reported	Continuous: recoded as dichotomous ≤2 or ≥ 3
Crime rate in the neighbourhood	Parent-reported	Re-coded as dichotomous: “disagreed/strongly disagreed”, and “agreed/strongly agreed”
Trust people in the community	Parent-reported	Re-coded as dichotomous: “disagreed/strongly disagreed”, and “agreed/strongly agreed”
Correlates specific to MVPA		
School physical activity policies	School-Administrator-reported	Binary: yes/no
Mother’s BMI	Parent-reported	Continuous: re-coded as dichotomous < 25 or ≥ 25
Father’s BMI	Parent-reported	Continuous: re-coded as dichotomous < 25 or ≥ 25
Sleep duration	Accelerometer measured	Continuous
Recreational screen time	Participant-reported	Continuous
Correlates specific to Sleep duration		
Recreational screen time	Participant-reported	Continuous
Moderate-to-vigorous-intensity physical activity	Accelerometer measured	Continuous: re-coded as dichotomous (< 60 min per day) or (≥60 min per day)
Correlates specific to ST		
Sleep duration	Accelerometer measured	Continuous
MVPA	Accelerometer measured	Continuous: re-coded as dichotomous (< 60 min per day) or (≥60 min per day)
Consumption of fast-food	Participant-reported	Re-coded as dichotomous: (eats fast food ≤3 times per week) or (eats fast food > 3 times per week)
Consumption of fried food	Participant-reported	Re-coded as dichotomous: (eats fried food ≤3 times per week) or (eats fried food > 3 times per week)
Consumption of fast food while watching television	Participant-reported	Re-coded as dichotomous: (does not eat fast food while watching television) or (eats fast food while watching television at least once per week)

*BMI*: Body Mass Index; *MVPA*: moderate- to vigorous-intensity physical activity; *ST*: recreational screen time

### Treatment of missing data

Overall, 103 participants (15%) were missing data on parental education and 149 participants (22%) had insufficient accelerometry data. Participants with missing data did not significantly differ in mean age (mean difference = 0.02 years; *p* = 0.7), or sex (chi-square = 1.31; *p* = 0.3). The proportion of participants missing data on the highest level of parental education did not differ from those with complete data (*p* = 0.7). To minimize loss of information, and potentially biasing the results due to excluding missing cases [39], multiple imputation by chained equations (MICE) was applied [40] using the R statistical Package, “mice” [41]. Missing values were multiply imputed (50 datasets) under the Missing At Random (MAR) assumptions [40], which were tested using recursive partitioning analysis [42], the Little MCAR’s test [43], and missing patterns analyses [40].

## Results

**Fig. 1:**
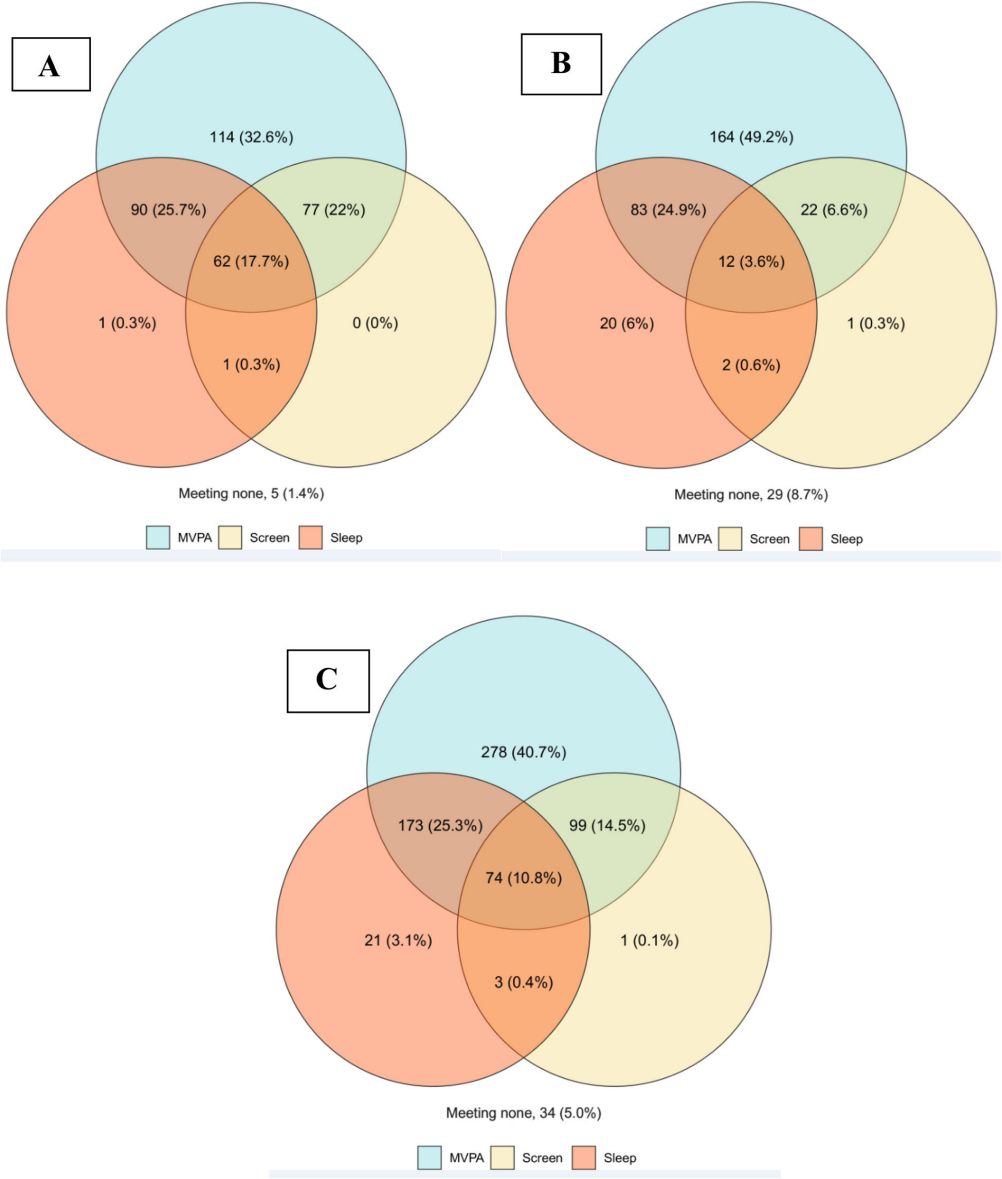
(*n* = 683): Venn diagrams showing the number and proportion of participants meeting no guidelines, the moderate-to-vigorous physical activity (MVPA), recreational screen time (screen), and sleep duration recommendations, and various combinations of the recommendations for the rural participants (panel 1**a**), urban participants (panel 1**b**), and the whole sample (panel 1**c**)

Table 2 presents descriptive characteristics of participants with complete data, stratified by urban and rural school location. Participants (52.9% girls) had a mean age of 10.1 ± 0.8 years. The mean MVPA minutes/day for both urban (82.9 ± 29.5), and rural (96.7 ± 31.8) children, were above the recommended 60 min/day. Average sleep duration was lower and average daily ST was higher than the recommended amounts for both groups. There were statistically significant differences for all descriptive characteristics between urban and rural participants except sex (χ^2^ = 0.8; *p* = 0.4). Venn diagrams presented in Fig. 1 show the proportions of participants meeting no guidelines, the MVPA, ST, and sleep duration recommendations, and various combinations of the recommendations for the rural participants (panel 1A), urban participants (panel 1B), and the whole sample (panel 1C). Figure 1 A shows that more rural (17.7%) compared to urban (3.6%) participants (Fig. 1 B) met all three 24-h movement guidelines. Figure 1 C illustrates that only 10.8% of the entire sample met all three of the 24-h movement guidelines, while 5% did not meet any of the recommended guidelines.

**Table 2:** Descriptive characteristics

Continuous variables	Mean (SD)				
	Total sample (*n* = 683)	Urban (*n* = 333)	Rural (*n* = 350)	t-value	*p*-value
Age (years)	10.1 (0.8)	10.2 (0.8)	10.1 (0.8)	2.5	0.01*
MVPA (minutes/day)	96.7 (31.8)	82.9 (29.5)	106.9 (29.5)	−9.3	<.0001*
Sleep duration (hours/day)	8.8 (0.8)	8.7 (0.9)	8.9 (0.7)	−2.5	0.01*
ST score (hours/day)	3.8 (2.4)	5.0 (2.3)	2.7 (1.9)	14.1	<.0001*
**Categorical variables**	**N (%)**			**chi-square**	**p-value**
Sex (% female)	683 (52.9)	333 (54.7)	350 (51.1)	0.8	0.4
MVPA (% meeting recommendations)	534 (89.0)	228 (77.2)	306 (97.7)	56.0	<.0001*
Sleep duration (% meeting recommendations)	530 (39.3)	227 (33.5)	303 (43.6)	5.5	0.02*
ST (% meeting recommendations)	662 (24.2)	318 (8.2)	344 (40.0)	85.4	<.0001*
Parents did not complete high school	431 (74.3)	172 (62.1)	259 (85.5)	48.8	<.0001*
Parents completed high school, some college	111 (19.1)	71 (25.6)	40 (13.2)		
Parents completed bachelors or higher degree	38 (6.6)	34 (12.3)	4 (1.3)		

Data are presented for participants with complete data

*SD* Standard deviation, *MVPA* Moderate- to vigorous-intensity physical activity, *ST* Recreational screen time

Meeting the recommendations is defined as ≥60 min/day for MVPA, ≤2 h/day for ST, and between 9 and 11 h per night for sleep

*= statistically significant at *p* < 0.05

Table 3 presents univariable correlates of meeting each of the three movement behaviours plus all of them combined. In the univariable analyses, four potential correlates had a marginal (*p* < 0.10) statistically significant association with meeting the MVPA guideline, eight correlates for meeting the ST guideline, and one each for the sleep duration guideline and all three 24-h movement guidelines. Results from multivariable analyses (Table 4) show that participants from urban schools were less likely to meet the MVPA guideline (OR: 0.19; CI: 0.07–0.52), ST guideline (OR: 0.34; CI: 0.12–0.93), or all three 24-h movement guidelines (OR: 0.21; CI: 0.09–0.52) than those from rural schools. Older children had lower odds of meeting the sleep duration guideline (OR: 0.75; CI: 0.59–0.95). Participants who engaged in sports were less likely to meet the ST guideline (OR: 0.47; CI: 0.29–0.78), and those reporting longer outdoor time had lower odds of meeting the ST guideline (OR: 0.62: CI: 0.51–0.75) or all three 24-h movement guidelines (OR: 0.67; CI: 0.53–0.86).

**Table 3:** Univariable correlates of meeting MVPA, sleep duration, ST and combination of all three Movement Guidelines (*n* = 683)

Variables	Estimate	SE	Odds Ratio	95% CI	p-value
Meeting MVPA Guideline					
Commute to school (ref: passive)	−0.54	0.36	0.58	0.29–1.18	0.13
BMI z-score	− 0.18	0.14	0.83	0.64–1.09	0.18
Outdoor time	0.16	0.10	1.18	0.96–1.44	0.12
Participation in sports (ref: did not participate)	0.44	0.38	1.56	0.73–3.30	0.25
Self-perceived health (ref: poor/fair)	0.67	0.33	**1.95**	**1.02–3.71**	**0.04**
Mother works (ref: < 15 h/week)	0.52	0.33	1.68	0.88–3.22	0.12
Father works (ref: < 15 h/week)	−0.74	0.33	**0.48**	**0.25–0.91**	**0.03**
Televisions in the house (ref: < 1)	−0.68	0.31	**0.50**	**0.27–0.96**	**0.04**
Functional cars at home (ref: < 2)	− 0.89	0.31	**0.41**	**0.23–0.76**	**0.004**
Number of siblings (ref: ≤2)	−0.02	0.29	0.98	0.55–1.74	0.95
Crime rate in the neighbourhood (ref: crime not a problem)	−0.44	0.30	0.65	0.36–1.17	0.15
Trust people in the community (ref: do not trust)	−0.08	0.30	0.92	0.51–1.66	0.78
School physical activity policies (ref: not present)	−0.14	0.73	0.87	0.18–4.22	0.86
Mother’s BMI (ref < 25 kg/m^2^)	0.28	0.29	1.32	0.74–2.36	0.34
Father’s BMI (ref < 25 kg/m^2^)	−0.36	0.31	0.70	0.38–1.27	0.24
Sleep duration	−0.00	0.00	1.00	0.99–1.01	0.81
ST (ref: not meeting)	0.00	0.06	1.01	0.89–1.14	0.93
Meeting Sleep duration Guideline					
Commute to school (ref: passive)	0.11	0.27	1.12	0.65–1.91	0.67
BMI z-score	0.11	0.09	1.12	0.95–1.32	0.19
Outdoor time	0.00	0.06	1.00	0.89–1.12	0.99
Participation in sports (ref: did not participate)	0.50	0.23	**1.66**	**1.05–2.60**	**0.03**
Self-perceived health (ref: poor/fair)	0.16	0.21	1.17	0.77–1.78	0.45
Mother works (ref: < 15 h/week)	0.00	0.19	1.00	0.69–1.45	0.99
Father works (ref: < 15 h/week)	−0.07	0.18	0.93	0.66–1.32	0.70
Televisions in the house (ref: < 1)	− 0.10	0.20	0.90	0.61–1.34	0.61
Functional cars at home (ref: < 2)	− 0.09	0.19	0.91	0.63–1.32	0.62
Number of siblings (ref: ≤2)	0.04	0.17	1.04	0.74–1.46	0.83
Crime rate in the neighbourhood (ref: crime not a problem)	0.00	0.18	1.00	0.70–1.42	0.98
Trust people in the community (ref: do not trust)	−0.13	0.18	0.88	0.62–1.24	0.46
MVPA (ref: not meeting)	−0.31	0.30	0.73	0.40–1.33	0.30
ST (ref: not meeting)	−0.03	0.04	0.97	0.90–1.05	0.45
Meeting ST Guideline					
Commute to school (ref: passive)	−0.73	0.43	**0.48**	**0.21–1.11**	**0.09**
BMI z-score	−0.00	0.10	1.00	0.82–1.22	0.98
Outdoor time	−0.50	0.09	**0.61**	**0.51–0.73**	**<.0001**
Participation in sports (ref: did not participate)	−0.67	0.23	**0.51**	**0.32–0.81**	**0.004**
Self-perceived health (ref: poor/fair)	−0.28	0.24	0.75	0.47–1.21	0.24
Mother works (ref: < 15 h/week)	−0.13	0.23	0.88	0.56–1.38	0.57
Father works (ref: < 15 h/week)	−0.19	0.21	0.83	0.55–1.25	0.37
Televisions in the house (ref: < 1)	−0.34	0.26	0.71	0.43–1.18	0.18
Functional cars at home (ref: < 2)	− 0.66	0.24	**0.52**	**0.32–83**	**0.006**
Number of siblings (ref: ≤2)	−0.28	0.21	0.75	0.50–1.14	0.18
Crime rate in the neighbourhood (ref: crime not a problem)	−0.38	0.22	**0.68**	**0.45–1.05**	**0.08**
Trust people in the community (ref: do not trust)	0.41	0.21	**1.51**	**1.00–2.28**	**0.05**
Sleep duration	0.00	0.00	1.00	0.99–1.00	0.73
MVPA (ref: not meeting)	0.83	0.55	2.29	0.78–6.73	0.13
Consumption of fast-food (ref: < 3 times per week)	−0.36	0.32	0.70	0.37–1.30	0.26
Consumption of fried food (ref: < 3 times per week)	− 0.51	0.22	**0.60**	**0.38–0.94**	**0.03**
Consumption of fast food while watching television (ref: < 3 times per week)	− 0.94	0.29	**0.39**	**0.22–0.68**	**0.001**
Meeting all three 24-Hour Movement Guidelines					
Commute to school (ref: passive)	−0.70	0.61	0.50	0.15–1.66	0.25
BMI z-score	0.03	0.13	1.03	0.79–1.35	0.81
Outdoor time	−0.39	0.12	**0.68**	**0.53–0.86**	**0.002**
Participation in sports (ref: did not participate)	−0.36	0.30	0.70	0.38–1.27	0.24
Self-perceived health (ref: poor/fair)	−0.13	0.32	0.87	0.46–1.65	0.68
Mother works (ref: < 15 h/week)	0.10	0.31	1.10	0.60–2.04	0.76
Father works (ref: < 15 h/week)	0.09	0.28	1.10	0.64–1.90	0.73
Televisions in the house (ref: < 1)	0.21	0.34	1.24	0.64–2.43	0.52
Functional cars at home (ref: < 2)	− 0.12	0.31	0.89	0.48–1.65	0.71
Number of siblings (ref: ≤2)	−0.26	0.29	0.77	0.44–1.35	0.36
Crime rate in the neighbourhood (ref: crime not a problem)	−0.49	0.30	0.61	0.34–1.10	0.10
Trust people in the community (ref: do not trust)	0.18	0.28	1.20	0.69–2.07	0.52

Data are presented from imputed datasets

*MVPA* Moderate- to vigorous-intensity physical activity, *BMI* Body mass index, *ST* Screen time, *SE* Standard error, *CI* Confidence interval

Bold indicates marginally statistically significant correlates at *p* < 0.10

Meeting the 24-h movement guidelines is defined as ≥60 min/day for MVPA, ≤2 h/day for ST, and between 9 and 11 h per night for sleep duration

**Table 4:** Multivariable correlates of meeting MVPA, sleep duration, ST and combination of all three Movement Guidelines (*n* = 683)

Variables	Estimate	SE	Odds Ratio	95% CI	p-value
Meeting MVPA Guideline					
Age	−0.39	0.24	0.68	0.42–1.09	0.10
Sex (ref: girls)	1.43	0.36	**4.18**	**2.08–8.41**	<.0001*
Parental education (ref: <high school, high school/some college)	−0.35	0.33	0.71	0.37–1.35	0.29
School location (ref: rural)	−1.69	0.50	**0.19**	**0.07–0.52**	0.002*
Self-perceived health (ref: poor healthy)	0.54	0.35	1.72	0.87–3.40	0.12
Father works (ref: < 15 h/week)	−0.66	0.35	0.51	0.26–1.02	0.06
Televisions in the house (ref: < 1)	−0.14	0.36	0.87	0.43–1.76	0.70
Functional cars at home (ref: < 2)	−0.59	0.34	0.56	0.28–1.09	0.09
Meeting Sleep Duration Guideline					
Age	−0.29	0.12	**0.75**	**0.59–0.95**	0.02*
Sex (ref: girls)	0.08	0.17	1.08	0.78–1.51	0.62
Parental education (<high school, high school/some college)	−0.13	0.21	0.88	0.59–1.32	0.54
School location (ref: rural)	−0.38	0.40	0.68	0.29–1.59	0.35
Participation in sports (ref: did not participate)	0.55	0.23	**1.74**	**1.10–2.76**	0.02*
Meeting ST Guideline					
Age	−0.03	0.14	0.97	0.75–1.27	0.84
Sex (ref: girls)	−0.25	0.21	0.78	0.52–1.17	0.22
Parental education (<high school, high school/some college)	−1.08	0.32	**0.34**	**0.18–0.64**	0.0008*
School location (ref: rural)	−1.10	0.49	**0.34**	**0.12–0.93**	0.04*
Commute to school (ref: passive)	−0.05	0.47	0.95	0.38–2.40	0.91
Outdoor time	−0.48	0.10	**0.62**	**0.51–0.75**	<.0001*
Participation in sports (ref: did not participate)	−0.74	0.25	**0.47**	**0.29–0.78**	0.003*
Functional cars at home (ref: < 2)	−0.62	0.27	**0.54**	**0.32–0.92**	0.02*
Crime rate in the neighbourhood (ref: crime not a problem)	−0.43	0.23	0.65	0.41–1.03	0.07
Trust people in the community (ref: do not trust)	0.34	0.23	1.41	0.90–2.20	0.13
Consumption of fried food (ref: < 3 times per week)	−0.32	0.25	0.73	0.45–1.19	0.20
Consumption of fast food while watching television (ref: < 3 times per week)	−0.68	032	**0.50**	**0.27–0.96**	0.04*
Meeting all three 24-Hour Movement Guidelines					
Age	−0.31	0.17	0.74	0.53–1.02	0.07
Sex (ref: girls)	0.07	0.26	1.08	0.64–1.80	0.78
Parental education (<high school, high school/some college)	−0.99	0.44	**0.37**	**0.16–0.87**	0.03*
School location (ref: rural)	−1.55	0.43	**0.21**	**0.09–0.52**	0.002*
Outdoor time	−0.40	0.12	**0.67**	**0.53–0.86**	0.001*

Data are presented from imputed datasets

*MVPA* Moderate- to vigorous-intensity physical activity, *ST* Recreational screen time, *SE* Standard error, *CI* Confidence interval, *SES* Socioeconomic status

*: statistically significant at *p* < 0.05

Models were adjusted for age, sex, SES, and school location

Meeting the 24-h movement guidelines is defined as ≥60 min/day for MVPA, ≤2 h/day for ST, and between 9 and 11 h per night for sleep duration

## Discussion

Results from this study revealed distinct differences in prevalences of meeting movement guidelines and related correlates between urban and rural children in Mozambique. To the best of our knowledge, this is the first study to compare accelerometer measured movement behaviours between urban and rural children in Mozambique. The observed differences in the prevalences and correlates of movement guideline adherence between urban and rural children supports our primary hypothesis and underscores the importance of including both groups for research and surveillance, especially in sub-Saharan Africa where the majority of the population still live in rural areas [16]. Sex (OR: 4.18; CI: 2.08–8.41, MVPA), school location (OR: 0.21; CI: 0.09–0.52, all three 24-h movement guidelines) and parental education (OR: 0.37; CI: 0.16–0.87, for all three 24-h movement guidelines) had medium to large effect sizes [44], suggesting moderate- to strong associations with adherence to movement behaviours.

The higher levels of MVPA among children in the present study differs from previous reports in high-income countries [1, 45, 46] but supports the results reported by Prista et al. [17] albeit from a sample of only rural Mozambican children, and demonstrates the key role that household, or utilitarian PA may be playing in these contexts. These findings merit discussing, debating and exploring public health messaging that encourages maintaining the high levels of MVPA for better health benefits. Furthermore, it may be useful for planners to include items that capture the various domains of PA (including utilitarian PA) when designing surveillance measurement instruments, enabling researchers to quantify and better characterize these sub-categories. The higher odds of boys than girls meeting MVPA guidelines is consistent with previously reported results from both high-income countries and LMICs [18, 47, 48], suggesting that it is not unique to one region or country, and may call for the need to deliberately evaluate the real or perceived contextual and cultural barriers to the equitable participation in MVPA for girls.

The fact that more rural children are adhering to movement guidelines than their urban counterparts may be related to the ongoing PA transition [49]. Accelerometer data obtained by Ojiambo et al. showed higher MVPA and lower sedentary behaviour among rural compared to urban Kenyan adolescents [18]. Systematic review evidence of studies from sub-Saharan Africa showed that children living in urban areas were more likely to engage in lower levels of PA, and higher sedentary behaviour than their rural counterparts [20]. Furthermore, an examination of secular trends in habitual PA in the city of Maputo, Mozambique revealed a general negative trend between 1992 and 2012 [50] and cited decreased household chores associated with economic and social transitions as likely contributing to the trend. Collectively, these results suggest that the urban environment itself (e.g. lack of walkability, reduced need for habitual PA, and safety concerns) may be detrimental to a healthy active childhood.

Consequences of urbanization (e.g. passive transportation) and modernity (e.g. availability of more screens) may be reducing the opportunities for habitual PA and increasing chances for sedentary pursuits among urban dwellers in Mozambique. Conversely, opportunities for engaging in utilitarian PA is likely preserved in rural areas where availability of space, lack of artificial lighting and less access to screens and may be helping children to attain more MVPA, longer sleep duration and have less ST. This observation is supported by our finding showing that only 1.4% of the rural participants failed to meet any guideline compared to 8.7% of the urban participants. In addition, our findings are consistent with results from previous research among children and youth elsewhere in Africa [18, 20, 48]. Movement behaviours for children and adolescents in our sample may also be influenced by other factors such as family affluence. For example, our results showed that children whose parents reported that they had ≥2 functional cars at home or those who reported frequent consumption of fast-food while watching TV, (both potential proxies of family affluence) had lower odds of meeting movement guidelines. Self-reported participation in sports and outdoor time had counterintuitive associations with adherence to the ST guideline and all three 24-h movement guidelines. It is possible that participants either over or underestimated times spent in these activities given the subjective nature of the instruments used, and the potential for social desirability and other associated biases. Alternatively, it is plausible that participants that could afford to participate in sports and have ample outdoor time in this context may be more affluent with less requirements for household and other chores, and are more likely to also afford ST opportunities.

Our finding showing average ST being higher than the recommended ≤2 h/day among both urban and rural children supports results from previous studies [3, 14], and may be indicative of the current pervasiveness of ST among children worldwide, even for those in LMICs, thus similarly exposing them to the associated health-related risks [51, 52]. Average sleep duration for the present sample (8.8 ± 0.8 h/night) was shorter than the recommended 9–11 h/night, but compares well to that reported for the whole ISCOLE sample (8.8 ± 0.9) [53], which was obtained using a similar protocol [32]. Although these findings only represent a snapshot in time, they add to the growing body of evidence [2, 54] suggesting secular trends for shorter sleep duration, albeit the mixed and conflicting evidence [2, 55]. Alternatively, shorter sleep duration may be inevitable in this population where school start times are generally early, and rural children may need to first complete household chores, walk long distances to school thus necessitating early wake up times. Given the higher average ST and shorter average sleep duration for the urban versus rural participants, it is plausible that modernity [54], and urbanization may be contributing to these findings.

The limitations of this study include its cross-sectional design which precludes inferences about directionality or causation. Associations found are limited to the list of available correlates and we also cannot exclude the potential confounding effects of unmeasured variables. Our sample is not necessarily representative, and for several variables, relied on self-reported data obtained by instruments whose validity and meaning in this context, especially for the rural population, has not been assessed. Because of missing data on key variables, multiple imputation was applied with potential of introducing bias. However, comparative analyses between complete data cases and imputed datasets do not support this potential limitation. Nonetheless, this study has important strengths that include our recruitment of a relatively large sample with both urban and rural participants. We objectively measured movement behaviours, and followed a rigorous and standardized study protocol. For example, research staff were trained and certified prior to data collection. Finally, our multilevel analyses accounted for the hierarchical nature of these data.

## Conclusion and future directions

The prevalence of meeting movement guidelines and related correlates differed between urban and rural children. On average, both groups had higher daily MVPA minutes, shorter sleep duration, and higher ST than recommended in the 24-h movement guidelines. It is important for future research to account for the environmental, contextual and cultural factors unique to LMICs and to urban and rural living. Further, public health messages should be tailored in these areas to promote and preserve higher MVPA, longer sleep duration, and less ST as the preferable way of life. Such a message could emphasize the additional benefits associated with exceeding the thresholds for MVPA given in the 24-h movement guidelines. Additionally, reducing excessive instead of excessing ST and optimizing sleep duration may be a priority over focusing on MVPA, in this population. Our study provides data that can be used to inform local policies and strategies and can serve as evidence supporting the need to include both rural and urban samples in studies and surveillance efforts, particularly in Africa.

## Supplementary information

**Additional file 1.** STROBE Statement—Checklist of items that should be included in reports of *cross-sectional studies*.

## Data Availability

The datasets used and/or analysed during the current study are available from the corresponding author on reasonable request.

## Abbreviations

BMI: Body mass index
CI: Confidence Interval
CIHR: Canadian Institutes of Health Research
DDFM = KR: Denominator degrees of freedom = Kenwardroger
ICC: Intraclass correlation coefficient
IDRC: International Development Research Centre
ISCOLE: International Study of Childhood Obesity, Lifestyle and the Environment
LMICs: Low-middle-income countries
MAR: Missing at random
MCAR: Missing Completely at Random
MICE: Multiple imputation by chained equations
MVPA: Moderate- to vigorous-intensity physical activity
NCDs: Non-communicable diseases
OR: Odds Ratios
PA: Physical Activity
SD: Standard deviation
ST: Recreational Screen Time
STROBE: Strengthening the Reporting of Observational Studies in Epidemiology
VIF: Variation inflation factors
WHO: World Health Organization
