# Supplementary material for: Prevalence and correlates of adherence to movement guidelines among urban and rural children in Mozambique: a cross-sectional study
Source: Int J Behav Nutr Phys Act. 2019 Oct 28;16:94. doi: 10.1186/s12966-019-0861-y (PMC6819612; doi:10.1186/s12966-019-0861-y)
Supplement: Supplementary file 1 — Additional file 1. STROBE Statement—Checklist of items that should be included in reports of cross-sectional studies. [file 12966_2019_861_MOESM1_ESM.doc]

STROBE Statement—Checklist of items that should be included in reports of ***cross-sectional studies***

|  | Item No | Recommendation |
| --- | --- | --- |
| **Title and abstract** | 1 | (*a*) Indicate the study’s design with a commonly used term in the title or the abstract: *The study design (cross-sectional) is included in the title of our manuscript “Prevalence and correlates of adherence to movement guidelines among urban and rural children in Mozambique: a cross-sectional study”.* |
|  |  | (*b*) Provide in the abstract an informative and balanced summary of what was done and what was found:  *We provide a balanced description of our methods (lines 38-46) and key results (lines47-53) in our abstract.* |
| Introduction | | |
| Background/rationale | 2 | Explain the scientific background and rationale for the investigation being reported:  *Research evidence show that insufficient physical activity, short sleep duration, and excessive recreational screen time among children are reaching alarming levels globally. Currently, there are little to no data describing prevalences and correlates of movement behaviours among children in low-middle-income countries (LMICs). The few available reports do not include both urban and rural respondents, despite the large proportion of rural populations in LMICs. Research has shown that PA can be negatively affected by childhood PA behaviours across the life-course can be heavily influenced by childhood experience.* |
| Objectives | 3 | State specific objectives, including any prespecified hypotheses:  *The purpose of the present study was to 1) compare the prevalence of urban and rural school children meeting 24-hour movement guidelines, and 2) examine correlates of meeting the 24-hour movement guidelines in a sample of urban and rural Mozambican children. We hypothesized that children attending rural schools would accumulate more minutes of moderate- to vigorous-intensity physical activity, have longer sleep duration and less recreational screen time compared to those from urban schools and that correlates of movement behaviours would differ between urban and rural children.* |
| Methods | | |
| Study design | 4 | Present key elements of study design early in the paper:  *We present the study design in the first two lines of our methods section (lines 114-116)* |
| Setting | 5 | Describe the setting, locations, and relevant dates, including periods of recruitment, exposure, follow-up, and data collection  *At least three urban schools were randomly recruited from each of three districts, using a list provided by the Ministry of Education to maximize variability in levels of neighborhood socioeconomic status (SES). Rural schools were conveniently recruited from a list provided by the district education office. Data were collected between August 2017 and May 2018.* |
| Participants | 6 | (*a*) Give the eligibility criteria, and the sources and methods of selection of participants  *Participants (9-11 year-old primary schoolchildren) for this cross-sectional study (n = 683) were recruited from 10 urban (Maputo, stratified by socioeconomic status) and 7 rural (Macia district) schools in Mozambique.* |
| Variables | 7 | Clearly define all outcomes, exposures, predictors, potential confounders, and effect modifiers. Give diagnostic criteria, if applicable  *Dependent variables: 24-hour movement guidelines (Sleep duration, recreational screen time, MVPA); predictors are listed in Table 1; covariates: age, sex, and residence (urban or rural) are included in our methods section.* |
| Data sources/ measurement | 8* | For each variable of interest, give sources of data and details of methods of assessment (measurement). Describe comparability of assessment methods if there is more than one group:  *Movement and body composition variables in this study were objectively measured as shown in the methods section (lines 131-153), screen time and potential correlates were obtained from validated items of questionnaires. We provide a reference for the sources of questionnaires, and correlates are listed in Table 1* |
| Bias | 9 | Describe any efforts to address potential sources of bias:  *We applied multiple imputation for missing data. We included covariates in multivariable models.* |
| Study size | 10 | Explain how the study size was arrived at:  *Sample size: Sample size calculations were partially guided by those done for ISCOLE. Assuming: 1) that participants would be recruited in clusters with an average of 25 students per school; 2) approximately 5.3% difference in obesity prevalence between urban (6.8%) and rural (1.5%) primary schoolchildren; and 3) at least 80% power, a total of 444 participants for both urban and rural (222 each) would be required. To account for the cluster sampling, we estimated a design effect of 1.3, resulting in a required sample size of 578; and anticipating approximately 10% of participants to have invalid and/or incomplete data, the recruitment target was 650 students.* |
| Quantitative variables | 11 | Explain how quantitative variables were handled in the analyses. If applicable, describe which groupings were chosen and why  *Please refer to measurement of variables section of our methods (pages 6-8) and Table 1* |
| Statistical methods | 12 | (*a*) Describe all statistical methods, including those used to control for confounding |
|  |  | (*b*) Describe any methods used to examine subgroups and interactions |
|  |  | (*c*) Explain how missing data were addressed |
|  |  | (*d*) If applicable, describe analytical methods taking account of sampling strategy |
|  |  | (*e*) Describe any sensitivity analyses  *Please refer to the methods and statistical sections of our manuscript as well as our treatment of missing data (pages 5-9).* |
| Results | | |
| Participants | 13* | (a) Report numbers of individuals at each stage of study—eg numbers potentially eligible, examined for eligibility, confirmed eligible, included in the study, completing follow-up, and analysed |
|  |  | (b) Give reasons for non-participation at each stage |
|  |  | (c) Consider use of a flow diagram  *This was cross-sectional study and we used multiple imputation to include all participants in this study.* |
| Descriptive data | 14* | (a) Give characteristics of study participants (eg demographic, clinical, social) and information on exposures and potential confounders |
|  |  | (b) Indicate number of participants with missing data for each variable of interest:  *Refer to Table 2 (descriptive characteristics and the section on treatment of missing data in our methods section.* |
| Outcome data | 15* | Report numbers of outcome events or summary measures |
| Main results | 16 | (*a*) Give unadjusted estimates and, if applicable, confounder-adjusted estimates and their precision (eg., 95% confidence interval). Make clear which confounders were adjusted for and why they were included: *Tables 2-5; Figure 1 provides key findings from the current study.* |
|  |  | (*b*) Report category boundaries when continuous variables were categorized: *These are reported in detail in the text of our methods as well as in Table 1.* |
|  |  | (*c*) If relevant, consider translating estimates of relative risk into absolute risk for a meaningful time period: *not applicable* |
| Other analyses | 17 | Report other analyses done—eg analyses of subgroups and interactions, and sensitivity analyses: *none done* |
| Discussion | | |
| Key results | 18 | Summarise key results with reference to study objectives:  *The observed differences in the prevalences and correlates of movement guideline adherence between urban and rural children supports our primary hypothesis and underscores the importance of including both groups for research and surveillance, especially in sub-Saharan Africa where most of the population still live in rural areas. Sex (OR: 4.18; CI: 2.08–8.41, MVPA), school location (OR: 0.21; CI: 0.09–0.52, all three 24-hour movement guidelines) and parental education (OR: 0.37; CI: 0.16–0.87, for all three 24-hour movement guidelines) had medium to large effect sizes, suggesting moderate- to strong associations with adherence to movement behaviours.* |
| Limitations | 19 | Discuss limitations of the study, taking into account sources of potential bias or imprecision. Discuss both direction and magnitude of any potential bias:  *The limitations of this study include its cross-sectional design which precludes inferences about directionality or causation. Associations found are limited to the list of available correlates and we also cannot exclude the potential confounding effects of unmeasured variables. Our sample is not necessarily representative, and for several variables, relied on self-reported data obtained by instruments whose validity and meaning in this context, especially for the rural population, has not been assessed. Because of missing data on key variables, multiple imputation was applied with potential of introducing bias. However, comparative analyses between complete data cases and imputed datasets do not support this potential limitation.* |
| Interpretation | 20 | Give a cautious overall interpretation of results considering objectives, limitations, multiplicity of analyses, results from similar studies, and other relevant evidence:  *On average, both urban and rural children had higher daily MVPA minutes, shorter sleep duration, and higher ST than recommended in the 24-hour movement guidelines. It is important for future research to account for the environmental, contextual and cultural factors unique to LMICs and to urban and rural living. Further, public health messages should be tailored in these areas to promote and preserve higher MVPA, longer sleep duration, and less ST as the preferable way of life. Such a message could emphasize the additional benefits associated with exceeding the thresholds for MVPA given in the 24-hour movement guidelines. Additionally, reducing excessing ST and optimizing sleep duration may be a priority over focusing on MVPA, in this population. Our study provides data that can be used to inform local policies and strategies and can serve as evidence supporting the need to include both rural and urban samples in studies and surveillance efforts, particularly in Africa.* |
| Generalisability | 21 | Discuss the generalisability (external validity) of the study results:  *Given the non-representativeness of our sample, and the narrow age range, the results from this study cannot be generalized to the entire population of Mozambique or other age groups of children.* |
| Other information | | |
| Funding | 22 | Give the source of funding and the role of the funders for the present study and, if applicable, for the original study on which the present article is based:  *Funding: Data collection for this study was partially funded by a doctoral award from the International Development Research Centre (IDRC). TM is supported by a Canadian Institutes of Health Research (CIHR) doctoral fellowship for allied health professionals. Study sponsors had no role in study design, data collection and analysis, decision to publish or preparation of this manuscript. Authors had full control of all primary data.* |

*Give information separately for exposed and unexposed groups.

**Note:** An Explanation and Elaboration article discusses each checklist item and gives methodological background and published examples of transparent reporting. The STROBE checklist is best used in conjunction with this article (freely available on the Web sites of PLoS Medicine at http://www.plosmedicine.org/, Annals of Internal Medicine at http://www.annals.org/, and Epidemiology at http://www.epidem.com/). Information on the STROBE Initiative is available at www.strobe-statement.org.
